# Notes and News

**Published:** 1904-07-16

**Authors:** 


### NOTES AND NEWS.

The Duchess of Albany opened the new wing of the Royal Richmond Hospital on July 8th. The building comprises out-patient and ophthalmic departments.

A severe epidemic of cholera is visiting Teheran. Several hundred fatal cases are reported daily.

We learn that some of the old wards in St. Bartholomew's Hospital are to be closed for the purpose of installing the electric light. The inference is that the authorities of St. Bartholomew's Hospital, now that they have secured the only new building they appear to desire, contemplate a long and restful period of structural inactivity. Else there can be no justification for the expensive undertaking they are commencing. It would seem that this is the beginning of a long list of patchwork and tinkering of the old wards which we predicted would be inevitable if any endeavour were made to maintain them, even with a semblance of efficiency.

The proceedings at the last meeting of the Swansea Hospital Board testify—if testimony be wanted—how very necessary it is that those who take an interest in hospitals, or, indeed, in any charitable institutions, should understand something of the chief principles of administration. At this one meeting we find the chairman of the House Committee declaring that the administration was "as extravagant as could be," whilst the chairman of the hospital quoted figures showing that the average expenditure was not extravagant. A member of the Board proposed, as a means of saving, that patients should be allowed to supply their own butter as formerly! He admitted this was a retrograde step. The result of the meeting was that, although the average number of beds occupied was 112½, it was voted necessary to curtail the number of available beds to 110 owing to the poverty of the hospital. Thus it appears evident that Swansea will be without sufficient hospital accommodation—a fact to be much regretted—and we cannot help thinking that, if organised and energetic means were taken, Swansea and its neighbourhood could be aroused to their responsibilities.

The death from the plague of an English medical officer is announced at Cairo.

The Metropolitan Hospital Sunday Fund collection has reached nearly £44,000.

Mons. Curie, the discoverer of radium, is to be the first occupant of the new chair of physics at the Paris University.

Lord Goschen distributed the prizes to the students of the medical school at Guy's Hospital on July 6th. The Dean of the College announced that a governor of the hospital, Mr. Robert Gordon, had promised to provide a museum, and that the staff of the hospital and medical school had decided to build a new lecture theatre and class-rooms adjoining this museum. The old buildings will then be pulled down and the site added to the hospital garden.

A largely attended reception in connection with the Strand district of the League of Mercy was held, at the invitation of Mrs. Carl Hentschel, lady president of the district, in the Prince's Room of the Criterion Restaurant. Mr. Carl Hentschel, president of the district, Dr. Norman Hay Forbes, a vice-president of the Tonbridge district, and others gave short addresses explanatory of the objects of the League. A well-attended old English fête was held in the old Rectory grounds, Enfield, by kind permission of Mrs. Barry, in aid of the Enfield district of the League of Mercy. The success of the fête was greatly due to the organising powers of Lady Somerset, lady president of the district, and the Rev. Harry Stevens, hon. secretary.

The immense number of entertainments held in the cause of charity, which have been a remarkable feature of the present London season, is causing considerable comment in the Press and elsewhere. One outcome has been an organised protest on the part of many of the leading artistes, who realise that their generous services have been considerably abused without very much benefit resulting to charities. These entertainments are mostly costly affairs, absorbing nearly all the takings, and yet leaving the patrons who have enjoyed an excellent entertainment under the impression that they are generous benefactors of some particular charity, and that they have discharged their duty towards the poor. In reality the only generosity exhibited is that of the already hard-worked artistes who give their services at the risk of actual loss to themselves. Nevertheless, undesirable for the majority of reasons as the charity entertainment mostly is, it may be argued with some truth that it is the only means by which help for the charities can be wrested from the selfish and thoughtless amongst the community. If that is so, then it would be a mistake to abolish them altogether. But it is to be hoped that those ladies who with the best of motives desire to devote their time, energies, and opportunities to help our charities by organising entertainments will be careful that the burden of the entertainments shall not fall upon those who have to seek a living for themselves, nor upon the already fully occupied officials connected with the benefiting charity. This also not infrequently happens.

